# Epigenomics as Potential Tools for Enhancing Magnitude of Breeding Approaches for Developing Climate Resilient Chickpea

**DOI:** 10.3389/fgene.2022.900253

**Published:** 2022-07-22

**Authors:** B. S. Chandana, Rohit Kumar Mahto, Rajesh Kumar Singh, Rebecca Ford, Niloofar Vaghefi, Santosh Kumar Gupta, Hemant Kumar Yadav, Murli Manohar, Rajendra Kumar

**Affiliations:** ^1^ Indian Agricultural Research Institute (ICAR), New Delhi, India; ^2^ Center for Planetary Health and Food Security, Griffith University, Brisbane, QLD, Australia; ^3^ School of Agriculture and Food, University of Melbourne, Parkville, VIC, Australia; ^4^ National Institute of Plant Genome Research (NIPGR), New Delhi, India; ^5^ National Botanical Research Institute (CSIR), Lucknow, India; ^6^ Boyce Thompson Institute, Cornell University, Ithaca, NY, United States

**Keywords:** chickpea (*Cicer arietinum* L.), epigenomics, DNA methylation, RNAi- RNA interference, mutagenesis, climate resilience, sustainability

## Abstract

Epigenomics has become a significant research interest at a time when rapid environmental changes are occurring. Epigenetic mechanisms mainly result from systems like DNA methylation, histone modification, and RNA interference. Epigenetic mechanisms are gaining importance in classical genetics, developmental biology, molecular biology, cancer biology, epidemiology, and evolution. Epigenetic mechanisms play important role in the action and interaction of plant genes during development, and also have an impact on classical plant breeding programs, inclusive of novel variation, single plant heritability, hybrid vigor, plant-environment interactions, stress tolerance, and performance stability. The epigenetics and epigenomics may be significant for crop adaptability and pliability to ambient alterations, directing to the creation of stout climate-resilient elegant crop cultivars. In this review, we have summarized recent progress made in understanding the epigenetic mechanisms in plant responses to biotic and abiotic stresses and have also tried to provide the ways for the efficient utilization of epigenomic mechanisms in developing climate-resilient crop cultivars, especially in chickpea, and other legume crops.

## Introduction

Chickpea is the world’s second most significant grain legume, produced primarily in the tropics, subtropics, and temperate zones. It is a self-pollinated annual crop with a genomic size of 740 Mbp (Arumuganathan and Earle, 1991). Chickpeas are prized for their high levels of dietary proteins (20–30%), carbohydrates (40%), fibers (3–6%), and lipids (3–6%) (Pushpavalli et al., 2015). In addition, it is also a good source of fiber, minerals, vitamins, lysine, and sulfur-containing key amino acids. It is a resilient crop that is well adapted to stressful situations and is a god’s gift to tropical farmers. Chickpea yields on average around 780 kg and can reach up to 2.5 tons per hectare. Various biotic and abiotic stresses have a negative impact on chickpea yield and productivity. The principal factors that limit chickpea production in farmers’ fields include abiotic (drought, salinity, heat, and cold stresses), biotic (insect pests including pod borers, aphids—*Aphis craccivora*, leaf miner, bruchid, etc. and diseases like Fusarium wilt, Ascochyta blight, grey mold, and root rots) stresses. Every year, abiotic stresses cause roughly 6.4 million tons of crop output losses, with soil salinity being primary environmental stress (Jha et al., 2014). Soil salinity is a serious barrier to crop output, and it affects almost 80 million hectares of arable land worldwide (Flowers et al., 2010). Since chickpea is a winter crop, it is subjected to low-temperature stress (0–15°C) during the reproductive stage, which results in a significant loss of flowers and hence pods, reducing output potential by 30–40%. High temperature stress chickpea in late-sown crops, primarily during reproductive and pod filling stages and drought stress at several stages of development; terminal dryness, combined with heat stress during blooming and seed filling can reduce yield up to 70% due to drought and heat stress (Kudapa et al., 2014). Climate change is expected to increase the frequency of temperature extremes (cold and heat), as well as inconsistency in rainfall patterns, necessitating the development of stress-tolerant and climate-resilient chickpea cultivars with region-specific traits that perform well under drought, heat, and/or low-temperature stress. Chickpea production in harsh settings has been improved through a variety of methods, including genetic variability, genomic selection, molecular markers involving quantitative trait loci (QTLs), whole-genome sequencing, and transcriptomics study. Biotechnological technologies have improved our understanding of the genetic basis of chickpea stress tolerance as well as plant responses to abiotic challenges, allowing us to build stress-tolerant chickpeas. The immensity of the current task of maintaining or improving productivity in the face of growing salinity to fulfill yield demands has been clearly recognized and leads to nearly 70% increase in crop production as a top priority (Amin et al., 2016; Hong et al., 2016). So far, Mendelian-based genetic approaches and the selection of heritable target DNA sequences have provided significant genetic improvements in many crop species. In addition, a greater understanding and ability to select beneficial epigenetic and epigenomic changes are proposed to encompass a more efficient and holistic strategy for crop improvement. This is because epigenomic mechanisms are central to governing many plant stress responses, including through cell-autonomous epigenetic switching. This enables the registration and memory of unpredictable genetic signals. The term epigenetics was coined by Waddington (1942) that is used as an intermediate factor between the genotype and phenotype. During gene expression studies, there are various heritable changes occurred due to mitotic and meiotic divisions and are not coded in the DNA sequence itself (Tsaftaris and Polidoros, 2000). Heritable changes in gene expressions are independent of DNA sequence variation and steadily congenital from one generation to another (Berger et al., 2009; Chen et al., 2010a). Variations in the heritability of epigenetic marks (changes) occur during mitotic and meiotic cell divisions. Transient epigenetic changes are not heritable, stout ones, and mitotically transmitted with genome imprinting (Spillane et al., 2001). The meiotically generated epigenetic changes are heritable across the generations without the need for the original stimulus until they are lost or erased. Epigenetic repression is limited to one locus—the genes next to *flowering locus C* (*FLC*) are affected by the cold temperature, for example, which is common for many genes in *Arabidopsis* (Kilian et al., 2007). The loss may be because of genetic change, may be spontaneous (unknown reason), or submission to ambient. Those differ from those that induced the initial epigenetic alterations. The mitotically heritable changes that are not kept through meiosis (epigenetic variation in somatic cells) are lost irrespective of the certitude that mitosis usually perpetuates genetic constitution, such as heterosis is explicated as any edge observed in hybrids. The reverberations of heterosis appear to follow a preferably uncomplicated epigenetic presumption in plants. In hybrids, if the gene is entangled in growth, such as photosynthesis, the plant expressed enhanced vitality (Ni et al., 2008). Heritable epigenetic changes are also referred to as “epialleles,” where the epialleles of a locus are identical DNA sequences but display different epigenetic states and hence have an influence on a range of phenotypes (Richards, 2006). These may be classified into three categories based on relative dependence on the genotype: 1) **Pure epialleles** that are solely epigenetic and independent of the genetic variations; 2) **Facilitated epialleles** that partially depend on genetic variation. An example of epiallele transposon is that undergoes DNA methylation spreading into a gene after the insertion of an adjoining transposon. That will be passed across the generations and the changes include both genetic and epigenetic differences; and 3) **Obligate epialleles,** which are directly influenced by the genetic variants and co-segregate with the methylation variants (Woo et al., 2007). Epigenetic variations include various post-transcriptional histone modifications. These modifications include the activity of non-coding RNAs, histone variants, and DNA methylation, which showed drastic changes in plants’ response to biotic or abiotic stimuli by changing the transcriptional profile. The memory-directed modifications lead to improved capacity to withstand future stresses (Berr et al., 2011). Thus, epigenetic mechanisms influence the accessibility of DNA to enzymes, resulting in a wide range of gene expression and mRNA splicing. These processes add complexity to the traditional genotype-environment interaction for understanding phenotype expression and development since they are differentially sensitive to the environment (Burggren, 2020). Epigenetic pathways have recently been found as a mediator of this interaction, allowing fast phenotypic diversity in a variety of settings (Burggren, 2016). Under the ongoing climatic changes in agricultural adaptability and resilience to environmental changes, epigenetics has emerged as a major crop development method, ultimately leading to the generation of stable climate-smart crops. This has paved the path for crop breeding to take advantage of epigenetic diversity. Even though epigenetics mechanisms have not been demonstrated in many crop species, most mechanistic investigations are from model plant species. Thus, there is a need to understand how epigenetic mechanisms are linked to mortality predictions as a result of climate change, which affects a wide range of fields, from environmental conservation to climate change mitigation efforts, and is expected to be more frequent under a climate-change scenario (Amaral et al., 2020; González-Benito et al., 2020). Understanding the roles of epigenetics inducing stresses including histone modifications and DNA methylations helps to uncover the mechanisms that regulate plant-stress interactions and conditions (Chinnusamy and Zhu, 2009). In this review, we have attempted to summarize epigenetic contribution to agricultural adaptation in response to climate change, epigenomic mechanisms, and describe several characteristics in plants, problems in utilization and hypothesize with an objective of the future potential use of epigenetic variations in developing more resilient chickpea crop the staple food legume.

## Understanding Epigenetics and Epigenomics in Plants as a Model System

Understanding of the epigenetic regulatory machinery and mechanism in plants has most notably been achieved in *Arabidopsis thaliana*, (http://www.arabidopsis.org) a model species. Studies on crops, particularly maize, have led to a better understanding and more deep insight into the epigenetic phenomenon (Brink 1956). The implications for maize epigenetic research in the post-genomics era are manifold and it would be difficult to expect future discoveries that are yet to come. However, explanations for an epigenetic regulatory mechanism to stress response in staple crops are still not explored (Arefian et al., 2019; Khan et al., 2019). Along with diverse and overlapping epigenetic regulatory pathways in the maize genome, it could be the most important area of paramutation research for exploration (Chandler et al., 2010). Various reports indicate the novel contributions of the model plant, which have generally been provided in epigenetics and epigenomics. It was the first report in which the discrimination between euchromatin and heterochromatin was explained well based on cytological analyses (Heitz, 1929). Studies in tomato and maize gave heritable changes in expressions related to individual alleles with alternative states, a phenomenon known as paramutation, an inter-allelic interaction that leads to heritable changes in gene expression through mitotic and meiotic routes (Pikaard and Mittelsten, 2014). Paramutation was first explained in maize (Brink, 1956) and furnished the proof for non-Mendelian epigenetic transmissions in plants. The frequent eventuality of entities with changed flower conformity was first explained in the 18th century by Carl von Linne. It was recently observed and reported that a silenced epiallele handled these changes, which contain DNA identical to an expressed allele (Cubas et al., 1999). The innovative exertion of interchangeable ingredients in maize was reported by Barbara McClintock and others (1940), which disclosed the innumerable connections between genetic conduct and epigenetic rules. McClintock (1950) worked and published various reports on the transposable element in maize.

Although various ways regulate the gene expression process in the eukaryotes, DNA methylation is a usual epigenetic process by which cells are used to have switching control of genes in the “off” mode. In the last few years, researchers have unfolded the mechanism of DNA methylation, which led to the fact that methylation is a significant constituent in several cellular mechanisms, including embryonic growth, genomic imprinting, X-chromosome inactivation, and preservation of chromosome stoutness (Phillips, 2008). There are several reported mechanisms in which methylation plays a critical role. Researchers have also connected faults in the methylation process to a series of catastrophic outcomes, including innumerable human diseases (Kelkar et al., 2009). So, we can conclude that these studies help in developing bioinformatics tools that have wide applications across species kingdoms, including chickpeas.

**FIGURE 1:**
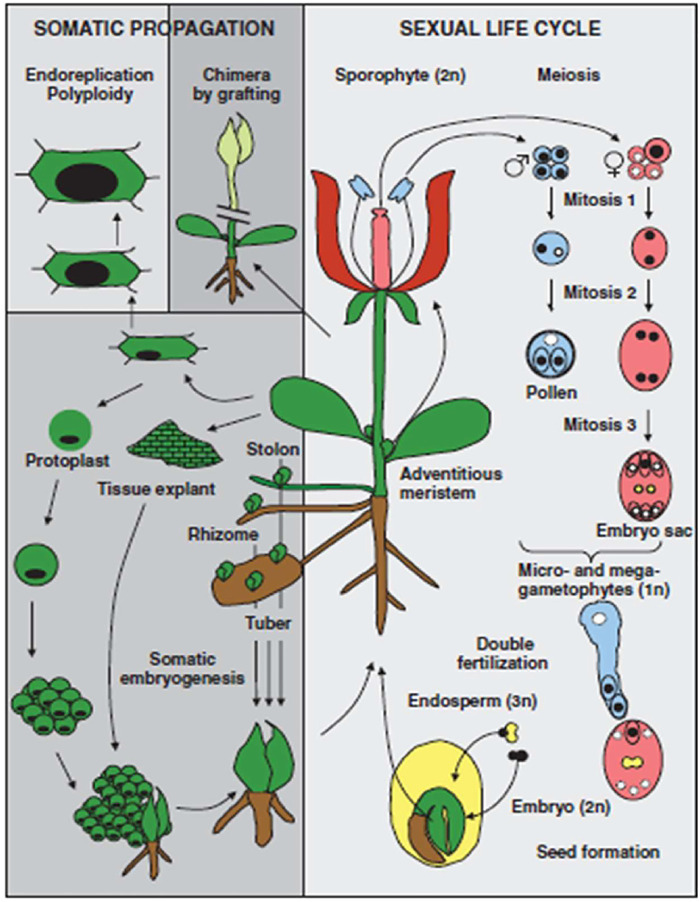
Unique aspects of the plant life cycle (Reproduced from Pikaard and Mittelsten, 2014).

In view of distinguishing differentiation between mammals and plants, it is important to consider the life cycle of plants. In mammals, fertilization is achieved by the fusion of two haploid cells produced by meiosis. However, in the case of plants, haploid (gametophyte) growth takes place that follows meiosis and precedes fertilization as presented in Figure 1. The male and female gametophytes are produced by mitotic divisions of the initial haploid meiotic products. In haploid gametophytes, loss of genetic or epigenetic information cannot be compensated for by information on homologous chromosomes. Unlike mammals, there is no evidence for a massive erasure of epigenetic marks during plant gametogenesis. Instead, repressive epigenetic marks in plant sperm and egg cells appear to be reinforced by specific trans-silencing RNAs produced in neighboring nuclei.

The first distinguishing feature to notice is that haploid (gametophyte) development begins with meiosis and continues after conception. There is a loss of genetic or epigenetic information in genetically and metabolically active gametophytes (haploids) that cannot be replaced by homologous chromosomes. In required genes, harmful mutation events are chosen in contrast. Plant gametogenesis, in contrast to mammalian gametogenesis, lacks proof for genome imprinting. Rather, certain trans-silencing RNAs originating in neighboring nuclei appear to be equipped in plant sperm and egg cells to suppress epigenetic changes. That may reflect the process of epigenetic changes that occur during meiosis in plants (Pikaard and Mittelsten, 2014). A second distinguishing trait of plants is the lack of a clearly defined germ line during early embryogenesis. Germ cells are formed in the later stages of plant development. Floral organisms emerge at this stage as a result of transformations from vegetative organs to progeny cells. Meiosis and gametogenesis take place in these cells. Thus, epigenetic changes acquired by meristematic cells in response to plant interactions in the presence of a specific environment have the potential to be passed to germ line cells, reviewed by Pikaard and Mittelsten (2014).

### Genetic Modifications in Model Plants for Epigenetics and Epigenomics

The arbitrary introduction of transgenes or transposable elements can be achieved in plant genomes by the process of chemical and or physical mutagenesis (Pikaard and Mittelsten, 2014). In the case of *A. thaliana*, it is very easy to identify homozygous mutants, which were identified amongst thousands of progenies of a single mutagen-treated plant. Marker gene is the basis to screen the putative mutants in epigenetic regulators. The promoter region of the *OsMYB91* gene was demethylated and rapid histone modifications at the *OsMYB9* locus in rice account for salinity resistance (Zhu et al., 2015). Increased *Asr1* and *Asr2* gene expressions have been observed during drought-resistance in tomato plants. The expression was enhanced because of the demethylation of putative regulatory and transcribed regions (González and Álvarez, 2013).

The advances in the production of transgenic plants have, therefore, adequately supported epigenetic and epigenomic research. In collaboration with forwarding genetics, another approach, i.e., reverse genetics emphasizing changing gene functions is also feasible. The development of mutants or utilizing transgene-starting RNAi has made an easy way to knockout or knockdown the expression of candidate epigenetic regulator homologs that were previously identified in other organisms. Once, a particular epigenetic mutant is characterized, restrained screenings are usually fortuitous for recognizing interacting constituents or alternative pathways, as observed with *Drosophila* (Elgin and Reuter, 2013) as well as with mouse (Blewitt and Whitelaw, 2013). However, because of the availability of components of epigenetic regulation and exhaustive assemblage of introduced mutations in almost every gene, schematic mutagenesis, and comprehensive instinctive dissimilitude*, A. thaliana* has developed as a model plant used for epigenetic studies based on various researches as presented in Table 1 (Pikaard and Mittelsten, 2014).

**TABLE 1:** Model plant *Arabidopsis thaliana* and their epigenetic regulation **(** Pikaard and Mittelsten, 2014 **)**

DNA Modification	Mutant name or gene	The Putative or Confirmed Function of Protein
*CMT3*	Chromomethyl transferase	DNA methyltransferase (mainly CHG and CHH)
*ROS3*	Silencing repressor	DNA glycosylase-domain protein, cytosine demethylation
*MBD10 Methylcytosine-binding protein*	Methylcytosine-binding domain protein	Methylcytosine-binding protein

## Mechanisms of Epigenetics and Epigenomics

**FIGURE 2:**
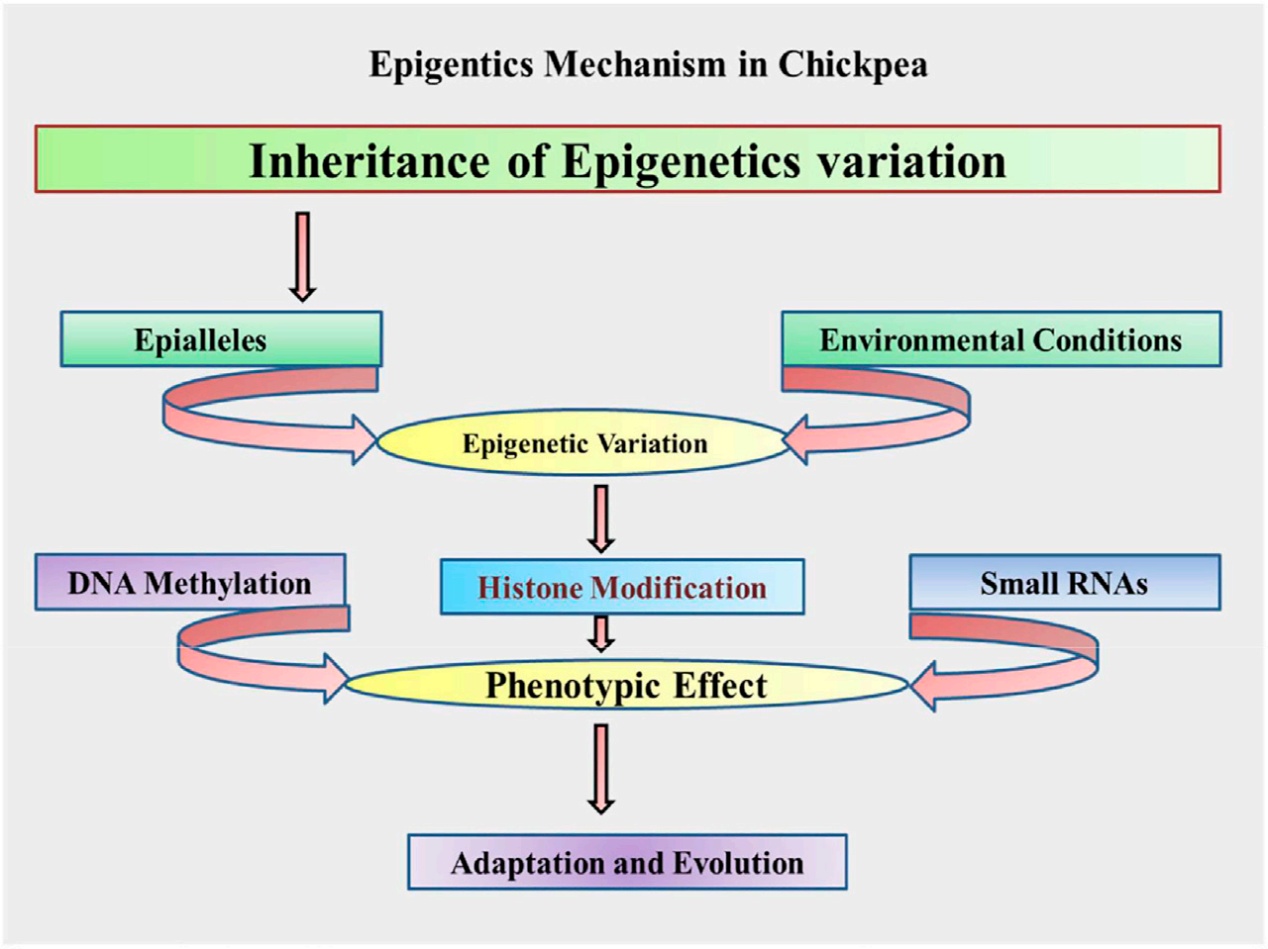
Proposed schematic mechanism/process of epigenetics in chickpea.

DNA methylation, histone/nonhistone alterations, and small RNA-mediated interference are the major mechanisms depicted in Figure 2, and explained as further in the following sections.

### DNA Methylation

DNA methylation is the addition of methyl group at the fifth position of carbon in the DNA molecule of cytosine. DNA methyltransferase carries out the post-replicative modification of DNA known as methylation using S-adenosyl-l-methionine (SAMe) as a methyl donor. Due to this modification, DNA conformation, protein interactions, and chromatin structure were changed, conclusively changing their functional states. Various important biological processes were driven by DNA methylation. These are cell developmental stages, X-chromosome inactivation, transposon tagging, genomic imprinting, and gene silencing. In the case of plants, DNA methylation usually occurs at the positions of CG, CHG, and CHH (H = A, C, or T) and intricate unique DNA methyltransferase, viz, DNMT3B, DNMT1, DNMT2, and DNMT3A (Jurkowska and Jeltsch, 2016). In the case of animals, DNA methylation patterns are associated with the origin, growth, developmental pattern, and progression of cancer (Jones and Baylin, 2007). DNA methylation patterns vary throughout the developmental differentiation in cells and tissues (Barlow and Bartolomei, 2014). This process is carried out by a family of DNA methyltransferase enzymes in eukaryotes. This helps in transferring the methyl groups from the methyl donor SAMe to the cytosine. The resulting 5-methylcytosine (5 mC) is often repressive and leads to gene silencing.

In plants including chickpeas, cytosine methylation is non-randomly distributed mostly to the repetitive regions that abundantly comprise transposable elements of the genome, centromeric frequencies, or multitudes of mute 45S or 5S rRNA gene recurrences. In addition, it further takes place by a handful of divergently controlled enhancers and within the protein-coding domains of tremendously conveyed genes in chickpeas (Zilberman et al., 2007). The eventual gene frame methylation is transformational preservation that plays a role during pre-mRNA splicing (Feng et al., 2010). DNA methylation plays immense contributions to various plant mechanisms, viz, gene silencing, imprinting of genes, plant immunity, escape from restriction enzymes, apomixis, etc. explained in the following sections.

### Long-Term Gene Silencing

The long-term silencing of the gene has been reported in DNA methylation patterns during reprogramming of seed differentiation and its functional relevancy in seed size and seed weight measurements in a large-seeded chickpea cultivar (JGK 3). The identified candidate genes involved in seed size/weight determination exhibited CG context hyper-methylation within the gene and manifold expression in JGK 3 provided insights into the role of DNA methylation in determining size, development, and weight of the seeds. The role of the RNA-dependent DNA methylation pathway has been shown by the gradual achievement of CHH-related DNA methylation in transposable elements (TEs) and by the elevated frequency of small RNAs in hyper-methylated TEs during the development of seed (Rajkumar et al., 2020).

### Imprinting of Genes

An epigenetic phenomenon that causes genes to be expressed in a parent-of-origin-specific manner is known as genomic imprinting. This is an inheritance process that is independent of the classical Mendelian inheritance and involves DNA methylation along with histone methylation without altering the genetic sequences. Genome imprinting has been reported with *Arabidopsis* that pushes forward to the rapid escalation of the endosperm, a desirable characteristic (Berger, 2006). This mechanism can apply to commercial crops and hybrids for their regeneration so that we can overcome the current limitations of plant breeding for the perpetual maintenance of hybrid vigor for generations. In the central cell of the female gametophyte, the Demeter molecule-DNA glycosylase domain quantity increases, causing DNA demethylation of the transposons because of a reduction in MET1 methyltransferase. Upon transcription of the transposons, a 24-nucleotide small interfering RNA (siRNA) is produced, which moves to the egg cell and causes imprinting of genes. Similarly, in the vegetative cells of the male gametophyte, DNA demethylation of the transposons occurs because of an increase in the diameter molecule, and upon transcriptions of these transposons, a 21-nucleotide small interfering RNA is produced that moves to the sperm cell and causes imprinting of genes (Bratzel et al., 2012).

Another study has dissected five legumes namely chickpea, soybean, alfalfa, pigeon pea, and lotus indicating the putative role of DNA methylation in the development of inheritable gene silencing and recognized potential DNA MTases (Garg et al., 2014). Based on the domain organization, MTases have been categorized into four subfamilies in legumes, viz, MET, CMT, DRM, and DNA nucleotide methyltransferases (DNMT2). The DNMT2 is a transfer RNA (tRNA) MTase, whereas the first three MTases are a class of DNA MTases. Structural comparative studies of all the known MTases in mammals and plants have assigned biological functions to these MTases (Jurkowski and Jeltsch, 2011). There are various reports in legumes related to the exhaustive gene expression assays of MTases that provide pieces of evidence of their important role in various developmental processes. During the plant life cycle and response to various abiotic stresses, the critical roles of MTases are continued until their survival (Benedito et al., 2008).

### Plant Immunity

Plant immunity is the built-in or catalyzed capability of plants to resist or avert a biological strike by pathogens. Molecules emancipated from pathogens are recognized by plant cell exterior sense organs; these receptors stimulate the specific showing cascades that facilitate to withstand the plant against pathogen infection. Roots activate specific tolerance mechanisms in response to elicitors such as molecular/pathogen-associated molecular patterns, (MAMPs/PAMPs), showing compounds (e.g., hormones), and plant defense activators (e.g., β-aminobutyric acid, BABA) (Zhang and Zhou, 2010). DNA methylation can have a critical role in plant immune responses to pathogens; for example, many defense genes in *A. thaliana are* modulated by DNA methylation starting defense reactions against *Pseudomonas syringae* pv *tomato* (Dowen et al., 2012).

**Escape from Restriction Enzymes:** Methylation-based modification interferes with the restriction process. It serves to protect bacterial chromosomal DNA against “self” restriction and is also responsible for the transient modification of those phages that escape restriction. It was reported in earlier studies that in bacteria, DNA methyltransferases provide a unique mechanism that handles the methylation of specific DNA sequences and is finally linked with epigenetic inheritance (Joseph and David, 2006). The restriction endonuclease recognizes a specifically marked DNA motif those are methylated by an analogs DNA methyltransferase when DNA methylation was primarily unzipped because of restriction-modification (R-M) forms. The R-M systems have been instigated as cellular protection, identifying incoming foreign DNA sequences (viral and another alien) for degradation. The methylation of foreign DNAs was based on specific recognition with related methylase of the same specification. The absolute methylation of the genome is enough to block double-strand DNA cut by the restriction enzyme when the restriction enzyme and its associated methylase both are expressed at levels in R-M systems. Owing to the cell demise that depreciated plasmid containing the EcoRV as post segregationally killing a plasmid comprising the type II R-M EcoRV pattern could not be substituted from the cells by a matchless plasmid (Nakayama and Kobayashi, 1998). The previous accomplishments have advised that R-M systems drive the features of selfish genes (Kobayashi et al., 1999; Kobayashi, 2001).

### Apomixis

Plants reproduce by sexual or asexual means and asexual reproduction in plants is carried out by cloning apomixes for sexually reproducing plants including chickpea, fertilization-independent seed formation is not possible because fertilization is a prerequisite for the embryo sac to develop into seeds. For apomictic plants, fertilization is unnecessary because the genes responsible for the fertilization-independent seed formation produce the apomictic seeds (Hand and Koltunow, 2014). Apomixis was proposed to have developed to enable plant species to propagate under adverse environmental conditions. The previous study has found that reactive oxygen species (ROS) are produced in excess in plants under stress and they possess innate systems for scavenging/detoxifying after they have done their job. Polyamines (putrescine, spermidine, and spermine) are low molecular weight, polycationic aliphatic molecules that are well known for anti-senescence and anti-stress effects because of their antioxidant properties (Kumar and Singh, 2016).

### Histone Modifications

DNA methylation, along with histone modifications that include histone acetylation, phosphorylation, methylation, and ubiquitination, contains the most identified epigenetic-associated mechanisms. Similar to DNA methylation, post-translational histone modifications do not possess the ability to influence the DNA nucleotide motif but might change its supply to the transcriptional system. Histone phosphorylation is another mechanism that is performed through histone modifications, often known for its creditability to DNA impairment in reaction to cell injury. The huge gene families in crop plants usually encipher histone-change enzymes by Berr et al. (2011), Deal and Henikoff (2011), and Lauria and Rossi (2011) and are explained in the following paragraphs.

Histone acetylation is carried out by histone acetyltransferases (HATs) enzymes, which mainly add acetyl groups to the histone tails and reduce positive charges and decrease the interaction of histones with DNA. HATs also facilitate transcription by enabling the DNA molecule more accessible to RNA polymerase II. Histone deacetylation is the reverse of histone acetylation and is carried out by Histone deacetylases (HDACs) enzymes. These HDACs remove acetyl groups from histone tails, increase the interaction of DNA, and repress transcription (Bird, 2007). Salinity and drought both are major environmental abiotic obstacles that cruelly affect overall global crop productivity and their nutritional quality (Sen et al., 2017). Plant-specific HD-Zip transcription agents are intricate in plant growth, development, and stresses. A novel HD-Zip (I) gene in chickpea, i.e*. CaHDZ12*, is expressed under water-deficit and salt-distress conditions. An improvement in the tolerance to osmotic stresses was observed in transgenic tobacco genotypes with over-expression *of CaHDZ12.* Silencing of *CaHDZ12* resulted in escalated sensitivity to salt and drought-distresses in chickpeas. Epigenetic changes like histone acetylation at the *CaHDZ12* promoting region have a critical role in distress-induced activation of this gene.

Histone methylation may cause the direct activation or repression of gene expressions and is carried out by histone methyltransferases (HMTs) having several classes including histone lysine methyltransferases (HKMTs), methylated lysine (k) residues, protein/arginine methyltransferase (PRMTs), and methylated arginine (R) residues. The trimethylation of histone H3 at lysine 4 (H3K4) is a specific region for transcription and demethylation to be carried on histone protein H3 to repress the transcription (Collins et al., 2019). Zentner & Henikoff (2013) reported that the addition of the methyl group from S-adenosyl-l-methionine (SAM) into lysine or arginine residues resulted in histone methylation. Histone methyltransferases (HMTs) are the enzyme that catalyzes this process (Bannister & Kouzarides, 2011). The DNA expression is changed through histone methylation by altering the engagement and the unbreakable controlling proteins adhered with the chromatin (Hyun et al., 2017). The histone lysine methylation occurs in mono-, di-, or tri-methylated forms, however, arginine methylation as mono- or di-methylated forms (Bannister & Kouzarides, 2011). The impact of histone methylation on DNA transcription depends on the numbers and to which residues methyl groups are getting added (Zentner & Henikoff, 2013). For example, methylations of H3K4, H3K36, and H3K79 are related to assiduous transcription, whereas the methylations of H3K9, H3K27, and H4K20 are related to tranquility (Black et al., 2012). The study conducted on DNA methylation and physio-biochemical analysis of chickpea in response to cold stress (CS) has reported that CS signals are converted as physiological changes as products of gene expression and are regulated by DNA methylation patterns (Rakei et al., 2015). The major roles of antioxidant enzymes (superoxide dismutase, catalase, ascorbate peroxidase, guaiacol peroxidase, and polyphenol oxidase) along with a noticeable ratio of changes in DNA methylation/demethylation patterns were often decisive factors in the preservation of cells against cold influenced oxidative distress.

### Enzymes Involved in Modifications of Histone Variants

In eukaryotic taxa, among the most conserved proteins include histone variants, linker histones, and non-histone proteins that are enciphered by highly superfluous gene families. The distinct categories of H2A and H3 histone variants, like animals also in plants based on their structure and function, have been identified (Henikoff and Smith, 2015). The physical properties of histone variants significantly affect their dynamic relations with DNA (Ingouff and Berger, 2010; Deal and Henikoff, 2011). The DNA damaged regions are discovered through phosphorylation of the H2AX variant and also assist during the recruitment of DNA repair proteins. A type of histone variant, H2A.Z exists mostly near the transcriptional start site of genes, most probably regulating transcription (Zilberman et al., 2008). The expression of this variant requires the initiative of an SWR1 chromatin-remodeling complex. Separation of DNA from H2A.Z comprising nucleosomes during heat stress is observed, which is followed by alterations in gene expression (Kumar and Wigge, 2010). During cell division, a particular histone H3 variant, i.e., CenH3 specifies the nucleosomes of centromeric regions and plays an important role in kinetochore assemblage, microtubule association, and chromosome segregation. H3.3, another histone variant having only a few different amino acids from its canonical H3 subunit, is predominantly found in regulatory regions. When specific linker histone proteins were downregulated as compensation, there is an upregulation of other histone variants. This results in a clear-cut phenotypic defective change with pleiotropy DNA hypomethylation (Jerzmanowski, 2007).

### Chickpea-Specific Histone-Modifying Enzymes

Several chickpea-specific histone-modifying enzymes as given further have been reported. The histone H2B is a core component of nucleosome that wraps up long compact DNA into chromatin, limiting DNA availability to the cellular mechanisms and using DNA as a template. Therefore, histones are an integral part of transcription regulation, chromosome stability, DNA repair, and DNA replication. A complex set of post-translational modifications of histones, known as histone code, and nucleosome remodeling help in regulating DNA accessibility. Histone H2B performs the molecular functions of DNA binding and protein heteromerization (https://www.uniprot.org/uniprot/Q9M3H6). It was reported that under heat stress conditions expression of six chromatin remodeling complex genes (*SWR1*) in diverse tissues of chickpea was based on nucleosome response through histone H2A.Z variants. A group of seven genes that are homologous to chromatin remodeling complexes (*SWR1*) of *Arabidopsis* was also identified in the chickpea genome. Three genes of chickpea homologs of photoperiod independent early flowering 1 (*PIE*), Actin associated protein (*ARP6*), two serrated leaves, and early flowering (*SEF*) for histone 2A variant-Z (H2A.Zs-a thermal sensor in plants) the three genes were analyzed for their appearances under heat distress and five diverse tissues. A significant role in chromatin remodeling complexes under heat stress conditions might be played by *CarPIE1* gene. The entire three histone CarH2A.Z variants acted as potential candidate genes for the characterization of their specific function (Chidambaranathan et al., 2016). These chickpea-specific histone proteins are summarized in Table 2.

**TABLE 2:** Chickpea-specific histone proteins (Chidambaranathan et al., 2016).

Gene Id	Protein ID	Groups	Protein size (aa)	MW(kDa)	pI
*LOC101514392*	XXP_004487028	III	150	15.95	10.75
*LOC101514067*	XXP_004487027.1	III	149	15.93	10.96
*LOC101514719*	XXP_004487029.1	III	148	15.75	10.73
*LOC101492287*	XXP_004493627.1	II	143	15.08	10.47
*LOC101500089*	XXP_004494603.1	III	146	15.42	10.35
*LOC101514555*	XXP_004494649.1	II	139	14.62	10.36
*LOC101489870*	XXP_004487625.1	I	135	14.06	10.05
*LOC101490207*	XXP_004487626.1	I	134	14.05	10.05
*LOC101507294*	XXP_004498649	IV	134	14.27	10.39
*LOC101489506*	XXP_004495152	IV	134	14.31	10.39
*LOC101497893*	XXP_004508547	IV	131	14.05	10.28

Furthermore, various forms of histone modifications and their sites along with effects on transcriptional activity of genes are summarized in Table 3.

**TABLE 3:** Histone alterations, their sites and impacts on the activities of transcription.

Alterations, sites, abbreviations	Impact on transcription
Acetylation of histone	
Histone 3 Lysine 4 acetylation (H3K4ac)	Activating/Permissive
Histone 3 lysine 9 acetylation (H3K9ac)	-do-
Histone 3 lysine 14 acetylation (H3K14ac)	-do
Histone 3 lysine 18 acetylation (H3K18ac)	-do
Histone 3 lysine 27 acetylation (H3K18ac)	-do
Histone 4 lysine 16 acetylation (H4K16ac)	-do
Histone 3 pan acetylation (H3ac)	-do
Histone 4 pan acetylation (H4ac)	-do
Methylation of histone	
Histone 3 lysine 4 methylation (H3K4me1)	Activating/permissive
Histone 3 lysine 4 dimethylation (H3K4me2)	-do-
Histone 3 lysine 4 trimethylation (H3K4me3)	-do
Histone 3 lysine 9 dimethylation (H3K9me2)	Repressive
Histone 3 lysine 9 trimethylation (H3K9me3)	-do
Histone 3 lysine 27 trimethylation (H3K27me3)	-do
Histone 3 lysine 36 trimethylation (H3K36me3)	Activating/permissive
Histone 3 lysine 79 methylation (H3K79me1)	Activating/permissive
Histone phosphorylation	
Histone 2A ubiquitination (H3S10ph)	Activating/permissive
Histone ubiquitination	
Histone 2A ubiquitination (H2Aub)	Repressive
Histone 2B ubiquitination (H2Bub)	Activating/permissive

### Role of Histone Modifications on Vernalization

The plant may continue to grow vegetatively through cell division during the cold period. When new seeds are produced, after the vernalization of the parent plant, the seeds are “reset.” The new plants they produce from the seeds will themselves have to go through their cold season before flowering. The key gene intricate in vernalization is referred to as FLOWERING LOCUS C or *FLC*. *FLC* encodes a protein known as a transcriptional repressor. It binds to other genes and stops them from getting switched on. These three genes *FT, SOC1*, and *FD* specifically regulate flowering in *Arabidopsis thaliana*, and show that the epigenetic status of *FLC* alters after a prolonged duration of cold. Experiments with mutated versions of epigenetic enzymes have shown that the changes in histone modifications at the *FLC* gene are critically important in controlling the flowering response. For example, there is a gene called *SDG27* that adds methyl groups to the lysine amino acid at position four on histone H3, so it is an epigenetic writer that is associated with a vigorous gene expression. The *SDG27* gene can be mutated experimentally so that it no longer encodes an active protein. Plants with this mutation have less of this active histone modification at the *FLC* gene promoter. They produce less *FLC* protein, and so are not so good at repressing the gene that triggers flowering. The *SDG27* mutants flower earlier than the normal plants. Cold weather induces protein in plant cells called VIN3 that works as chromatin and can bind to the *FLC* promoter. When *VIN3* binds to the *FLC* promoter, it alters the local structure of the chromatin instead of how tightly chromatin is wrapped up, making it often available to other proteins. Often, opening up chromatin leads to an increase in gene expression. However, in this case, *VIN3* attracts yet another *FD* (FLOWERING DETERMINATE) enzyme that can add methyl groups at position 27 on lysine residue amino acid of histone H3 protein. This modification represses gene expression and is one of the most important methods that plant cell uses to switch off the FLC gene. Following cold weather, the cells in *Arabidopsis thaliana* produce a long RNA, which does not code for a protein called *COLDAIR.* The *COLDAIR* non-coding RNA is present, particularly in the FLC gene. When localized, it binds to the enzyme complex that creates the important repressive mark at position 27 on histone H3. *COLDAIR,* therefore, acts as a targeting mechanism for the enzyme complex. From these data, we can see that flowering plants use some of the same epigenetic machinery as many animal cells. These include histone protein alternations, and the utilization of long non-coding RNAs to target these changes. Earlier, it has been inferred that destabilization of the cells is a consequence of global DNA hypomethylation through DNMT1-depletion led, which ultimately leads to the production of aneuploids (Barra et al., 2012). In the case of aneuploids (45, XO; 46, XX; and 47,XXX) expression of DNA methyltransferase 1 and DNA methylation enzymatic gene showed a positive association with inactive X chromosomes (Rajpathak and Deobagkar, 2017).

### Role of Histone in Epigenetic Regulation in Antiviral Innate Immunity

To deduce and establish the critical role of histones in epigenetic regulation during the process of viral innate immunity, there is a great need for better grasping of these complicated interactions through the epigenetic lens, which may have therapeutic opportunities in the clinic. A grasping of the parts played by the key epigenetic controllers—chromatin remodeling and histone alterations—in atonements of chromatin candidness in the process of host defense against virus, how the RNA alteration m6A (N6-methyladenosine) influences basic features of hostvirus interplaying and conclusions with subsequent orchestrations for better understanding about epigenetic regulations in host and viruses’ contaminations are required (Xiao et al., 2021).

### Non-Histone Proteins and Their Roles

Similar to other eukaryotes, non-histone chromosomal proteins are also found in plants, which may assist epigenetic gene regulations, including HMG proteins. The HMGB family of proteins is the foremost assayed and varied subgroup of proteins in plants, members of whom differentiate in the level of expression, localization, style, and inter-playing with DNA along with other proteins. The partial sub-functionalization of individual family members results from mutation and abnormal expression showing their role in developmental stages and response to various stress stimuli (Pedersen and Grasser, 2010). The structure-specific recognition protein (*SSRP1*) indirectly contributed to the demethylation of DNA the recognized genes in the female gametophyte’s central cell (Ikeda et al., 2011). Yuan et al. (2011) have reported that the structure, assembly, and rejection of cohesion seem to be highly protected. There are only limited family members in plants that might have specific functionalities. It was also found that the defective meristem silencing 3, which was involved in *de novo* (*DMS3/IDN1*) is required for transcription of DNA-dependent RNA polymerase V (RNA Pol V) (Haag and Pikaard, 2011). This plays a very important role in the establishment of *RdDM* (RNA-controlled DNA methylation) proteins (Varshney et al., 2019a). The various mutant screens related to epigenetic regulators are the sites of *REPLICATION FACTOR C1* and *REPLICATION PROTEIN A2* (*RPA2*) (Elmayan et al., 2005; Kapoor et al., 2005). The role of this protein related to stem cell and meristem repair is also revealed when chromatin mutants having increased phenotypes and mutants having topoisomerase homolog *MGOUN* (MGO) functional loss were merged (Graf et al., 2010). This signified that many other non-histone proteins involved with DNA will also act as direct or indirect epigenetic regulators.

### Nucleosome-Organizing Proteins

Short-term or long-term modifications in the nucleosomes' positioning and their connection with DNA were always required for replication, transcription, recombination, and maintenance. As a result, vigorous mechanisms on the chromatin amend DNA or protein modifications, incorporate alterations in nucleosome’s possession and, constitution, along with the attainability of the DNA to variegated proteins (Pikaard and Mittelsten, 2014).

### Chromatin-Remodeling Complexes

Becker and Workman (2013) have reported that chromatin remodeling can be used for the relocation or dissociation of nucleosomes. It was first reported in yeast and was named after the respective processes that ATPases such as the *SWI/SNF* complexes have influenced the mutants. Many similar complexes were also reported in plants (Jerzmanowski, 2007). The functional information for very few putative chromatin remodelers has been got through genetic screens, the first recognized being DECREASE IN DNA METHYLATION1 (*DDM1*). The function of *DDM1* included genome-wide decreased activity of methylation of DNA and H3K9me2, activating repetitive elements for transcription, and downregulation of many such genes. Therefore, the mutants for *ddm1* exhibit severe defects in developmental and morphological growth that may reach an extreme in future generations. The amalgamation of epimutations and insertional mutations induced through reactivated transposons is the main reason behind the gradually diminished fitness of ddm1 mutants. In ddm1 mutants, epigenetic information is permanently deleted and can be restored through backcrosses with wild-type plants as epigenetic patterns at several loci, mainly because of the outcome of *de novo* methylation (Teixeira et al., 2009). *DDM1* also shows *in vitro* ATP protease nucleosome moving initiative (Brzeski and Jerzmanowski, 2003). Mutants deficient in *DDM1* and linker histone H1 originate when cytosine methylation deficiency occurs in ddm1 mutants (Zemach et al., 2013) signaling that the requirement of *DDM1* is essential for the repair of methylation mechanics to discover DNA in nucleosomes consisting of core and linker histones. The defective RNA-mediated DNA Methylation 1 (*DRD1*) and *CLASSY 1* (*CLSY1*), extremities of the *SWI2/SNF2* family found in *Arabidopsis*, are unique to the plant kingdom including chickpea, and play a peculiar part in RNA-induced DNA methylation. Four additional *SWI2/SNF2* derived proteins namely; BRAHMA (BRM), MINUSCULE (1, 2), and SPLAYED (SPD) are intricate in the RNA-led DNA methylation (Sang et al., 2012). Other than ATPases, quintessence parts of *SWI/SNF* remakes are also reported in plants, inclusive of one SNF5 homolog (BSH), two *SWP73* homologs, and many *SWI3* family members (*AtSWI3 A-D*) (Jerzmanowski, 2007). However, their direct contributions to plants are still unexplored. However, it has been unraveled that *SWI3* interplays with RNA binding proteins lead to RNA-directed DNA methylation (Zhu et al., 2012).

The role of histone protein for drought and yield index (DYI) in chickpeas was studied, and it was disclosed that the development of functional molecular tags derived from the cis-regulatory sequence components of genes is crucial for their deployment and identification of several conserved non-coding SNPs (CNSNP). Among those, the two made-up natural haplotypes and alleles are derived from a histone H3 protein-coding gene and its transcriptional regulator NAC transcription factor (TF) anchoring the major QTLs and trans-acting eQTL controlling drought yield index (DYI) in chickpea (Sharma et al., 2019).

### RNA-Mediated Interference

RNA interference (RNAi) is a technique in which tiny RNA molecules are combined with other molecules to target homologous DNA regions. They bring together the agents that alter chromatin, resulting in heterochromatin formation and gene suppression. Pre-transcriptional gene silencing can stop transcription from happening. As a result, DNA methylation at genomic locations corresponding to complex siRNA or miRNA is catalyzed by an enzyme complex. RNA interference in chickpea and other legume crops has been found to have a large number of drought-responsive miRNAs. In response to salt stress, 259 miRNAs were shown to be differentially expressed in the root tip of chickpea during drought and salinity stress, which were also seen in other legumes such as soybean root apex (Khandal et al., 2017). TIR1 (TRANSPORT INHIBITOR RESPONSE 1), an auxin receptor, and AUXIN RESPONSIVE FACTOR 10 (ARF10) and ARF16 are targets of miR393 and miR160 (Chen et al., 2011). Overexpression of miR160 causes unregulated cell division and a loss of gravity sensing at the root tip during primary root development (Mallory et al., 2005). MiR164 inhibited auxin signaling for lateral root initiation by targeting the transcription factor NAC1. ARF6 and ARF8, which are positive regulators of adventitious root growth, were targeted by miR167 (Gutierrez et al., 2009; Guo et al., 2005). Comparative miRNA expression profiling of *Medicago truncatula* (Medicago) in the root tip and elongation zone, as well as root-forming callus and non-root forming callus, revealed 107 miRNAs from 44 families expressed in these tissues and predicted conservation of some of the miRNA/target relationships seen in other species (Eyles et al., 2013). Overexpression of MiR396 in Medicago roots inhibits cell-cycle gene expression and limits root development (Bazin et al., 2013). miRNA expression analysis in normal soybean roots, as well as comparisons between phosphate-starved and phosphate-sufficient soybean roots, revealed some new miRNA/target interactions (Xu et al., 2013). In Arabidopsis, an increase of miR393, miR397b, and miR402 expression occurs under dehydration and salt stress, according to a study. miRNA has a vital function in controlling root growth under abiotic stresses (Ding et al., 2009; Lu et al., 2014). Drought stress increases the expression of miR398a/b and miR408 in the Medicago root (Trindade et al., 2010) and miR169g in rice roots (Zhao et al., 2007). In Arabidopsis, mi RNA165/166 regulates root development by targeting transcripts of leucine-zipper family proteins (Singh et al., 2014). In Medicago, overexpression of miR160 altered root development and nodule number (Bustos-Sanmamed et al., 2013). In another work, epigenetic modulation of drought stress in chickpea was investigated. They notably researched MicroRNAs (miRNAs), non-coding RNAs that have been identified as significant controllers of gene performances like BHLH23 operating at post-transcriptional stages, which are implicated in tolerance to water constraints, as well as extra abiotic distress. BHLH23 transcription factor, which encodes for low copper levels, was found to be downregulated, and another drought stress-responsive gene, APETALA2/Ethylene Response Factors (ERF/AP2), was found to have a lower expression profile in miR408 over-expressed chickpea plants when compared to vector control plants after stress treatment (Hajyzadeh et al., 2015).

Methylation of cytosine and modifications of histone plays an important role in the gene regulatory mechanisms of genes responsible for epigenetic changes in plants. These modulations serve as gene regulators during transcriptional activities. Post-transcriptional modifications in context to epigenetic regulation occur through targeted degradation of mRNA. Finally, translational repression occurs and post-transcriptional gene silencing (PTGS) of mRNAs acts as a defense molecule against several pathogens. These are viruses, bacteria, fungi, molds, and transgene (Ruiz and Voinnet, 2009; Vazquez et al., 2010). The small RNAs play an active role in transcriptional and post-transcriptional gene silencing in plants (Chapman and Carrington, 2007). The actions of these miRNAs or siRNAs in plants have a resemblance to eukaryotic biogenesis (Shabalina and Koonin, 2008). However, multiple pathways have been involved in the duplication and sub-functionalization of genes guiding miRNA or siRNA-mediated processes in plants as follows (Herr, 2005; Baulcombe, 2006).

i. Biogenesis process for the miRNAs that are complementary to the targeted sequences;
ii. A ropeway in which a miRNA activates the origin of secondary trans-acting siRNAs with no complementarities to the starting miRNAs;
iii. A route for siRNA-intervened abasement of infringed viral RNAs or transgene RNAs along with
iv. A route for siRNA-intervened methylation of DNA, transcriptional mute of transposons/viruses, and other genes.

Variegation of the core machinery for siRNA biogenesis along with function pinpoints the evolutionary process of the various small RNA suppressing mechanisms in plants (Vazquez et al., 2010). Plants, like fission yeast (*S. pombe*) and nematodes (*C. elegans*), also make use of RNA-dependent RNA polymerases in dsRNA production. *Arabidopsis* genome enciphers six unique RdRPs. The plant Dicer produces diversified sizes of small RNAs, viz, miRNAs of 21 nt (*DCL1*), or siRNAs of different sizes 23–24 nt (*DCL3*), 22 nt (*DCL2*), or 21 nt (DCL4). These diverse siRNAs differ in size but overlap in functions, due to their relatedness to variegated AGO protein that contains 10 extremities in *Arabidopsis* (Vaucheret, 2008).

### Plant-Specific RNA-Directed DNA Methylation

Various proteins, such as AGO, Dicers, and RdRPs, were employed in a kind of permutations for accomplishing *de novo* methylation that occurs during the process of RdDM. It is initiated by the methyltransferases (DNA) of DRM grade. The exhaustive recruitment processes of *DRM2* in DNA that takes place are still not conspicuous. The process is undertaken in green algae before plants prominently produce RNA Pols IV, and V, the complex configurations of RNA Pol II (Luo and Hall 2007; Tucker et al., 2010). RNA Pols II, IV, and V each have 12 basic parts in *Arabidopsis*, nearly half part of that is often for the above three explained polymerases and distorted by the interchangeable genes (Ream et al., 2009). The genes that originated through the reoccurrence of RNA Pol II subunit genes, bolstered with sub engaged for certain subunits enciphered the subunits that are specific to RNA Pols IV or V (Ream et al., 2009; Law et al., 2011).

Paramutation is an interaction between alleles of a gene in such a way that an allele is heritably affected by another allele. This phenomenon is explained nicely through the booster-1 (b-1) site in maize (Brink, 1956). A para mutable (B-l) allele (active allele) after getting affiliated with a para-mutagenic (B’) allele (inactive allele) becomes a paramount (B-l*) allele. Uniform DNA sequences for the two alleles at the b-1 locus are found but vary in the system of methylation (DNA). Para mutant allele itself displays as para mutagenic and is unchanged through one or more following generations. However, most alleles are neither para mutable nor para-mutagenic.

The above discussed various mechanisms and processes governing epigenetics can be adopted in chickpeas and presented through a schematic flow diagram depicted in Figure 2.

## Epigenetic and Epigenomic Studies in Chickpea

As presented in the Table 4, the epigenetic studies in biotic and abiotic stress response, DNA methylation is a crucial component in gene assertion control. The DNA methylation status in seven resistant and susceptible cultivars of chickpea for *Fusarium oxysporum* f. sp. was determined using the methylation-sensitive amplified polymorphism (MSAP) assay and 27, 468 DNA fragments were obtained, each of which represented a recognition site cleaved by one or both isoschizomers amplified using selected primers (Mohammadi et al., 2015). They showed DNA methylation patterns in leaves, stems, and roots from both controlled and inoculated plants, and found extensive cytosine methylation modifications in pathogen-treated/infected plants, but none in controls. Heterologous expression of WRKY40 promoter and its transcriptional regulation via epigenetic alteration controls the fusarium stress resistance. This expression aids in the prevention of bacterial infections spreading due to resistance (Chakraborty et al., 2018). The important function of the WRKY40 transcription factor in the susceptibility of chickpea to *Fusarium oxysporum* f. sp. ciceri race 1 (Foc1) and resistance to this strain (WR315) has been demonstrated. In a controlled and Fusarium-affected environment, the histone changes in two chickpea genotypes were evaluated using immunoblotting and real-time PCR techniques. In the process of resistance interaction with Foc1, location-specific Histone three lysine nine acetylation, a positive signal of transcription, becomes reinforced at the WRKY40 promoter. In Foc1-infected susceptible plants, the H3K9 Ac is reduced at the WRKY40 promoter. The salt tolerance mechanism in chickpea was studied using an epigenetic approach in FLIP 97-43C (salt-tolerant) and FLIP 97–196C (salt-susceptible), which aids in the discovery of proteins that regulate photosynthesis, distress responsiveness, and protein assimilation (Arefian et al., 2019).

**TABLE 4:** Epigenetic studies related to biotic and abiotic stresses in chickpea.

S. No.	Biotic/Abiotic stress tolerance through an epigenetic mechanism	References
1	Salt tolerance mechanism in chickpea	Arefian et al. (2019)
2	Mechanism of drought stress	Khan et al. (2019)
3	The study on DNA methylation pattern Development and differentiation of seed size	Rajkumar et al. (2020)
4	Mechanism of salt tolerance in chickpea	Khandal et al. (2017)
5	Physio-biochemical analysis of chickpea in response to cold stress	Rakei et al. (2015)
6	Chickpea drought, water, and osmotic stress	Kaur et al. (2002) and Elkoca et al. (2007)
7	DNA methylation patterns in cultivated chickpea to understand the regulation of gene expression in different organs	Bhatia et al. (2018)
8	Drought and salinity resistance by an epigenetic mechanism in chickpea	Sen et al. (2017)
9	DNA methylation and epigenetics mechanism on physio-biochemical analysis of chickpea in response to cold stress	Rakei et al. (2015)
10	The epigenetic mechanism to heat stress in chickpea	Chidambaranathan et al. (2016)
11	Role of epigenetics in drought yield index	Sharma et al. (2019)
12	Chickpea, drought water and osmotic stress	Kilian et al. (2007)

### Epigenetics Studies in Other Legumes

The cytosine residues in the DNA of pea root tips subjected to water deficit were investigated to see if there was a link between environmental stress and DNA methylation. Two complementary approaches were used to assess DNA methylation: (i) immunolabeling with a monoclonal antibody against 5-methylcytosine, and (ii) MSAP (Methylation-Sensitive Amplified Polymorphism) to see if methylation and demethylation in response to water deficit could be linked to specific DNA sequences (Labra et al., 2002).

Plant microRNAs were investigated in beans (Dela et al., 2019). They are generally transcribed in transcripts with a single microRNA precursor, which is processed by DICERLIKE 1 and associated proteins to produce a short RNA, which is then incorporated into an AGO-containing protein complex to direct silencing of an mRNA with a complementary target sequence. Certain microRNA loci have several precursor stem-loop structures, encoding multiple microRNAs in a single transcript that is one-of-a-kind example in which the evolutionarily conserved miR398a is encoded in the same transcript as the legume-specific miR2119. Other legumes showed the same dicistronic configuration as the common bean. The role of small RNAs in reaction to water stress was investigated in *Phaseolus vulgaris*, and it was discovered that mature miR398 and miR2119 are repressed in response to water deficit, but that they are functional since they target the mRNAs for CSD1 and ADH1, respectively. The down-regulation of miRNA with the consequences of upregulation of CSD1 and ADH1 genes in common beans and possibly in other legumes respond to water deprivation (Naya et al., 2014). In the case of cowpea, the homology search was used to predict miRNAs and their targets. Real-time quantitative PCR was used to confirm the identified cowpea miRNAs in the leaves and roots of drought-stricken cowpea plants. Target gene prediction reveals that a group of miRNA target genes is implicated in metabolic pathways associated with physiological changes caused by drought stress. We looked at the expression levels of some key genes involved in physiological responses to drought stress and discovered that differences in their expression levels corresponded to the various drought responses of drought-sensitive and drought-resistant cowpeas (Shui et al., 2013).

The legume miR1514a activates phasiRNA by modulating a NAC transcription factor transcript. MicroRNAs have been identified as post-transcriptional regulators implicated in stress responses in recent investigations. In Phaseolus vulgaris (common bean), miR1514a is a legume microRNA that is activated in response to drought stress and has varying levels of accumulation in roots during water deficit in two cultivars with different drought-resistance phenotypes. The role of miR1514 in the regulation of a NAC transcription factor gene via phasiRNA synthesis during response to drought has been reported in case of soybean (Sosa et al., 2017).

## Integrating Epigenomics With Omics Approaches for Biotic and Abiotic Tolerance in Chickpea

Many biotic and abiotic stress tolerance gene(s) in plants have been discovered through recent advances in next-generation sequencing (NGS) (Garg et al., 2016). Integration of omics-generated data from several platforms, such as transcriptomics, which is coupled with proteomics, and finally, metabolomics, is essential to close the genome-to-phenome gap in agricultural plants. These platforms and their data enable to identify the certain phenotypes based on genetic contribution (Choi, 2019). The use of the omics strategy to gather genomic information to influence various biological processes, as well as the discovery of differentially expressed genes in various environmental situations and positional cloning. This strategy can also be utilized in the targeted region with an mRNA or protein shift to uncover the role of connected genes associated with the trait of interest (Su et al., 2019). A comparison of salt stress generated FLIP 97-43C (salt-tolerant) and FLIP 97–196C (salt-susceptible) was undertaken to understand the salt tolerance mechanism in chickpea, which resulted in the identification of proteins regulating photosynthesis, distress responsiveness, and protein absorption (Arefian et al., 2019). The researchers discovered 134 proteins that were expressed differently in the extracellular matrix and during the dehydration response. During the comparative proteomics investigation of JG-62, these proteins were discovered in a variety of biological roles (Bhushan et al., 2007). Through a targeted metabolomics approach, Khan et al. (2019) identified key upregulated metabolites such as allot in, l-proline, l-arginine, and l-histidine, as well as downregulated metabolites such as alanine, choline, gamma-aminobutyric acid, and phenylalanine, that were differentially expressed under drought stress conditions. From sugars to organic acids, a total of 48 distinct metabolites were discovered. Under salt stress, 28 biogenic amino acids were expressed in chickpea cultivars with varying salt tolerance (Dias et al., 2015). The specified compounds were quantitatively analyzed using modern metabolomics techniques and GC and LC were integrated into a triple quadrupole mass spectrometer (GC-QqQ-MS and LC-QqQ-MS). As a result, the reports for drought tolerance mechanisms in chickpea genotypes, omics techniques, and crop production management were shown to be the best and most cost-effective.

The cultivated chickpea has a narrow genetic base (Varshney et al., 2013) and phenotypic plasticity (Berger, 2006). It is difficult to locate the stress-responsive and undeniably tolerant gene(s), especially when plants accept cross-talk to react to many concurrent distresses (Tuteja, 2007). The physiological and genomic screening revealed that there was a wide range of genetic differences among and within the tolerant and sensitive genotypes for salinity tolerance. For example, cold was included in tolerant-1 and inhibited in tolerant-2 during gene profiling using microarray aquaporin genes for drought, salinity, and homology-based induction for salinity, heat, and environmental stress (Mantri et al., 2007; Kotula et al., 2015; Kaur et al., 2008). Several haplotypes and significant numbers of alleles associated with agronomic parameters in chickpeas have been uncovered using genomic resources (Varshney et al., 2019a). Fine mapping of ‘QTL-hotspot’ for drought tolerance-related features for the region of 7.74 Mb–300 kb and chickpea bin mapping were done using genotyping-by-sequencing and skim-sequencing, respectively (Varshney et al., 2014; Jaganathan et al., 2015). A huge range of resources, including genetic, genomic, and transcriptome resources, have been created over the last decade as a result of developments in various NGS technologies, transforming the chickpea crop from an orphan to a genomic-rich resource (Varshney et al., 2009; Kudapa et al., 2014; Agarwal et al., 2016; Mashaki et al., 2018). In chickpea breeding projects, next-generation sequencing, high-throughput genotyping technologies, and cost-effective omics methods are critical. Translational genomics in crop breeding has been made possible by the availability of molecular markers, sequencing platforms, genotyping assays for low-to-high density, quality check panels, draught genome assemblies, and sequence-based genetic variants (Roorkiwal et al., 2014; Thudi et al., 2016; Varshney et al., 2019a; Rasheed et al., 2017; Varshney et al., 2021).

### Integration and Impacts of Next-Generation Sequencing Technologies for Improving Chickpea Epigenetics

The advances in next generation sequencing (NGS) technologies have a lead impact on epigenomic research. The arrival of NGS technologies has introduced powerful sequencing methods–like, ChIP-Seq--to interrogate whole-genome histone modifications, improving on the conventional microarray-based method (ChIP-chip). More importantly, studies of DNA methylation and histone modification using NGS technologies have yielded new discoveries in plant biology too. The recent developments of third-generation sequencing technologies have shown promising results of directly sequencing methylated nucleotides and having the ability to differentiate between 5-methylcytosine and 5-hydroxymethylcytosine. The importance of 5-hydroxymethylcytosine remains largely unknown, but it has been found in various tissues. 5-hydroxymethylcytosine was particularly enriched at promoters and in intragenic regions (gene bodies) but was largely absent from non-gene regions in DNA from human brain frontal lobe tissue. The presence of 5-hydroxymethylcytosine in gene bodies was more positively correlated with gene expression levels. The importance of studying 5-methylcytosine and 5-hydroxymethylcytosine separately for their biological roles will become clearer when more efficient methods to distinguish them are available (Ku et al., 2011).

In contrast to histone modification profiling, a wide variety of approaches have been developed to profile DNA methylation utilizing next-generation sequencing platforms. Approaches to profile DNA methylation genome-wide can be broadly divided into those that rely on methylation-dependent enzymatic restriction, methyl-DNA enrichment, and direct bisulfite conversion (Fouse et al., 2010; Harris et al., 2010). Individual methods can also be combined to increase the resolution or efficiency of a single method. For example, a combination of MeDIP-seq and MRE-seq to profile both the methylated and unmethylated fractions of the genome (Maunakea et al., 2010)*.*

Advances in next-generation sequencing (NGS) technology have considerably curtailed sequencing costs resulting in the evolution of genotyping methods from individual marker-to whole-genome sequencing-based genotyping. This has resulted in the development of large-scale genomic resources, including genome sequence assemblies, re-sequencing of a few thousand lines, high-resolution genetic maps, and a range of low-to high-density genotyping platforms. These genomic resources were used to find alleles and haplotypes linked to chickpea agronomic traits (Varshney et al., 2019b). Genetic diversity, population structure, domestication patterns, linkage disequilibrium, and the untapped genetic potential for chickpea improvement have all been studied using whole-genome re-sequencing (WGRS) (Varshney et al., 2019a). Varshney et al. (2021) conducted a study on molecular diversity in chickpeas to describe genomic diversity across cultivated and wild progenitors. They found chromosomal segments and genes that show signatures of selection during domestication, migration, and improvement. The chromosomal locations of deleterious mutations responsible for limited genetic diversity and decreased fitness were identified in elite germplasm along with the superior haplotypes for improvement-related traits. They found targets for purging deleterious alleles through genomics-assisted breeding and/or gene editing. We can use this sequence information to find the DNA methylation regions that are responsible for biotic and abiotic tolerance in the chickpea in future breeding approaches.

### Advanced Technologies Assisted Epigenomics as Key Tools for Climate Resilient Chickpea

**FIGURE 3:**
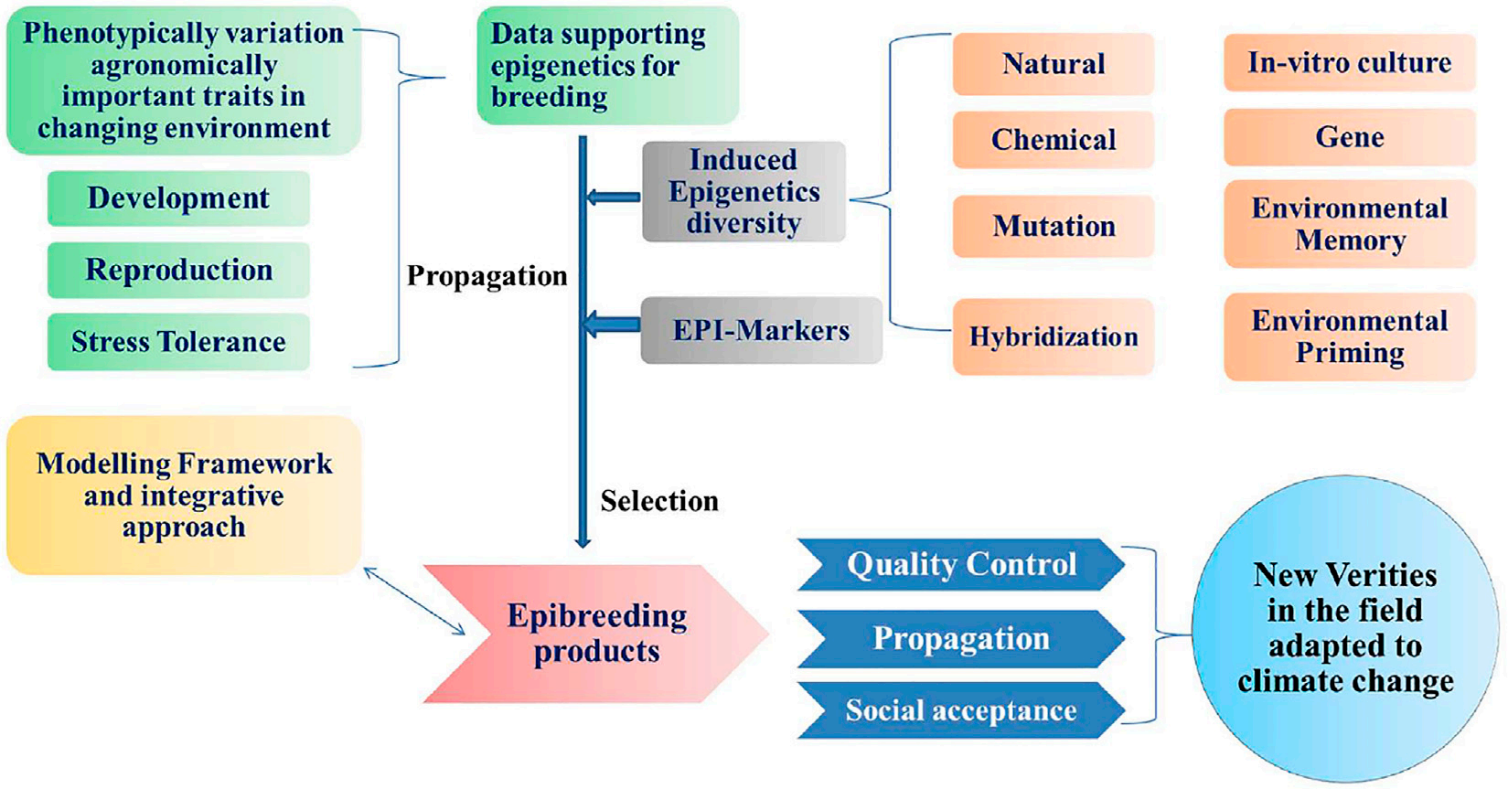
Schematic integrated epigenetic data and tools will lead to epi-bred crops and new varieties in the field adapted to climate change (Modified from Kakoulidou et al., 2021).

Epigenetic mechanisms have proven to have a role in enhancing plants’ resilience to environmental stresses, targeting varied traits, thus, giving a significant tool in breeding for climate-resilient crops. Epigenetic variation was applied for crop improvement to increase soybean yield (Raju et al., 2018). RNAi silencing of the plant-specific gene MutS HOMOLOG1 (*MSH1*) paved the way for the growth of epi-lines with variability for the arrangement of yield-associated traits in glasshouse and field trials. New epigenetic diversity indicted by MSH1 oppression was transmitted for at least three progenies. Similarly, the identification of epigenetic variations and regulatory mechanisms in chickpea plants, which impact important agronomic traits, can be exploited for epigenetic breeding for climate-resilient crops. The following schematic presentation as depicted in Figure 3 for the development of epigenetic data and tools will lead to breed of newer ep-breeds and varieties in the field and adapted to climatic changes.

Crop plants often have challenges of biotic and abiotic stresses, and they adopt sophisticated ways to acclimate and cope with these through the expression of specific genes. Changes in chromatin, histone, and DNA mostly serve the purpose of combating challenges and ensuring the survival of plants in stressful environments. Epigenetic changes, due to environmental stress, enable plants to remember a past stress event in order to deal with such challenges in the future. This heritable memory, called “plant stress memory”, enables plants to respond against stresses in a better and more efficient way, not only for the current plant in prevailing situations but also for future generations (Chao et al., 2021). Stress memory can also be described as a mechanism to enhance the resilience of crop plants (Walter et al., 2011), and the accumulation and changes in proteins (structural and regulatory) as transcription, translation, and transduction, which play an important role in the growth, development, and memory mechanisms of plants for stress resistance (Bruce et al., 2007; Janmohammadi et al., 2015, Marcos et al., 2018b). As the epigenetic modifications are environmentally accelerated, the phenotypic changes are mostly a reflection of a specific environmental interaction, and the changes adopted by the plant for a specific period may become permanent and heritable for future generations (Chinnusamy and Zhu, 2009; Verhoeven et al., 2010). Stress memory in plants is enhanced by up and downregulated sRNAs (miRNAs, siRNAs) they mainly downregulate negative regulators, upregulate positive regulators and regulate plant hormones, reactive oxygen species (ROS), and transcriptional factors in response to abiotic stress (Banerjee et al., 2017; Zhang et al., 2019).

Plant stress memory is achieved through the coordination of physiological, translational, transcriptional, and epigenetic activities in response to stress (Kinoshita and Seki, 2014; Hu et al., 2016). These regulatory processes can occur at any stage of plant development and are primarily controlled by epigenetic changes to phenotypically remodel for environmental stress (Rehman et al., 2015; Gallusci et al., 2017). Genetic diversity has been reduced as a result of intense breeding, and now epigenetic variation has arisen as a viable option for crop genetic improvement (Gallusci et al., 2017). There have been many developments for the quantification of epigenetic variations and their impact on the growth and development of plants, leading to improved yield and quality, and ultimately, this has opened another avenue for breeders to breed desirable agronomic characters successfully (Cortijo et al., 2014)]. In epigenetic modifications, DNA methylation plays an important role in gene regulation, expression, and stabilization (Lang et al., 2017). Various enzymes (DNA methyltransferase), targeted under different plant regulatory pathway systems, take part in the process to catalyze DNA methylation for a better and quicker response against biotic and abiotic stresses (Zhang et al., 2018). Epigenetic modification has the ability to memorize the event over a long time as a plant molecular memory and the ability to respond rapidly with heritable phenotypic characteristics as an inheritance system against environmental fluxes. Some extreme abiotic stress treatments can lead to plant genome reorganization (Klumpp et al., 2004; Molinier et al., 2006) but few reports are indicating that short-term stress causes a large number of genomic mutations (Lämke and Bäurle, 2017; Cruzan et al., 2018). More evidence supports the speculation that plant stress memory is mainly regulated by epigenetic pathways (Tang et al., 2014), which means changing the expression pattern of the entire genome to form a rebalanced genome expression system, without changing the genome sequence (Habu et al., 2001; Madlung and Comai, 2004).

Epigenetic variants can be produced through chemical treatment (5-azacytidine), epigenomic editing (TALENs), zinc finger nucleases, and the CRISPR/Cas system to counter biotic and abiotic stresses. There is an immense amount of care needed because targeted genes may be involved in complex and multiple pathways, which may cause complex and unexpected pleiotropic effects (Garg et al., 2015; Hung et al., 2018). All of these methods have tremendous scope for the creation of epigenetic variations (Kapazoglou et al., 2018) For successful breeding through epigenetic memory, it is necessary that variations should be inherited. DNA methylation changes and histone modifications are often reset during meiosis, meaning stable inheritance of the epigenetic mark is a problem in successful breeding goals achievement (Danchin et al., 2019).

Several reports revealed a correlation between the regulation of gene expression and changes in chromatin modifications in plants during stress exposure (Pandey et al., 2016). Epigenetic processes are crucial adaptive mechanisms that change the expression of genes in a heritable way without accompanying changes in DNA sequences (Chinnusamy and Zhu, 2009). Thus, heritable, but simultaneously reversible alterations in the transcriptional potential of cells are possible (Chen et al., 2010b). In a eukaryotic cell, the structure and function of chromatin depend upon several regulatory epigenetics. In plants such as *Arabidopsis thaliana*, *Oryza sativa*, and *Zea mays*, it was recently reported that hyper- or hypomethylation of DNA induced by abiotic stimuli can modulate the expression of stress-responsive genes (Wang et al., 2009; Van Dijk et al., 2010) mechanisms, including DNA methylation and histone modifications (Sahu et al., 2013). Epigenetics has a major role in symbiotic nitrogen fixation in chickpea, reported and experimentally proved by epigenetic regulations in the development of symbiotic root nodules of legume plants–EPISYM project. They have discovered that epigenetic regulations, involving plant DNA (de)methylation and small interfering RNA (siRNA) populations are essential to produce nitrogen-fixing nodules. They hypothesized that epigenetic regulations play an important role in gene expression reprogramming associated with nodule differentiation.

### Quantitative Epigenetic Models for Complex Traits

The accurate genetic assays of epigenetic variability and mapping of epigenetic quantitative trait loci (epiQTL) facilitated by the development of epi-RILs in Arabidopsis have provided a close association amongst epialleles and phenotypic characteristics. The epigenetics research in plants has taken a leap by employing epigenome-wide association study (EWAS) and epi-genotyping by sequencing (epi-GBS). Thus, chickpea improvement programs eventually can utilize the huge avenues provided by quantitative epigenetics to assay the contribution of epigenetic variability in trait control. Further, molecular breeding of important crop plants can be potentially facilitated by epigenome-editing tools, such as clustered regularly interspaced short palindromic repeats/CRISPR-associated protein 9 (CRISPR/Cas9), for locus-specific DNA methylation (Vijay et al., 2020).

Several statistical methods exist to detect epigenetic variations and their impact on the phenotype or epiQTLs. The significance and accuracy of epiQTLs identification are affected by several factors like recombination, transgressive segregation, instability of epialleles, and parent-off-origin effect. These factors may create confounding effects during epiQTLs analysis and result in false positives or false negatives. To deal with these interrupting factors, Johannes and Colomé-Tatché (2011) advocated as most suitable population generated from the crosses between epigenetic isogenic lines. Tal et al. (2010) deduced covariances amongst kinships owing to epigenetic transmission and environmental effect and modeled the number of events for epigenetic reset amongst generations and environmental inductions, and estimated the heritable epigenetic variance along with the rate of transmission. Furthermore, the necessity of multiple replication-wise testing due to the occurrence of several false positives is the critical bottleneck of quantitative genetics. During genome-wide recognition of epigenetic variation to encounter the bottleneck of false positivity a statistical model was developed (Jaffe et al., 2017). The missing heritability contributed by epigenetic variability may be studied by employing these models but leaving aside epigenetic-induced phenotypic variability (Roux et al., 2011). Furthermore, another improved model was proposed to predict the proportion of genetic variation and estimate phenotypic variation explained by epigenetic variation and their effects on phenotypic values along with the interaction of genetic effects (additive and dominant) and epigenetics (Wang et al., 2012).

### Quantitative Aspects of Epigenetics

Epigenetic markers are randomly present with high frequencies in the genome and are stably inherited through generations. The identification of epiQTLs is facilitated by these characteristics that permit the utilization of epigenetic markers. Unlike QTLs where polymorphism for the DNA nucleotide sequence occurs, epiQTLs are epigenomic loci that differ in cytosine methylation patterns that control phenotypic variability. Cortijo et al (2014) recognized major epiQTLs explaining 60–90% heritability by employing ddm1-derived *Arabidopsis* epi-RILs for quantitative traits root length and flowering time. These epiQTLs were observed to be useful for artificial selection and were reproducible. Furthermore, on the basis of inheritance and recombination events using mutagenic accumulation lines epigenotype map (E-map) was constructed and 99.9% of epialleles were observed to be stable (Hofmeister et al., 2017). In another study employing methylation-sensitive amplified fragment length polymorphism (MS-AFLP) and retro transposon epimarkers epiQTLs for seven agronomic characters were recognized in Brassica (Long et al., 2011). During varied developmental, environmental, and transgenerational states highly stable epigenetic marks were observed. In Sorghum, employing MSAP genotyping approach and 122 methylation polymorphic loci E-map harboring methylation hotspots, was constructed. In soybean, localization of methyl QTL (QTLs associated with DNA methylation) was facilitated by employing differentially co-segregated methylated regions (DMRs) in RILs. Thus, the crop where genetic variability is the negligible stable inheritance of epialleles through the generations makes it a potential controller of phenotypic variability. However, till date very limited number of EWAS have been accomplished in plants, but employing somatic clones (diverse for mantled abnormality and oil yield), a locus MANTLED was localized where hypomethylation in LINE retro transposon pushes the alternate splicing and premature termination epigenetic modification associated with a mantled abnormality in oil palm (Ong-Abdullah et al., 2015).

## Methods to Modify the Plant Epigenome

Besides the genes inclusive of its genome, the genetic constitution of any organism also contains its epigenome including methyl classes to specific sequences within the DNA that work as epigenetic marks to minimize transcriptions, and thus the expressions of the linked genes. Several methods applied to change the plant’s epigenome contained mutagenesis, carcinogenesis, plant tissue culture, CRISPR/Cas9 genome editing, and RNAi, which are explained below.

### Role of Mutagenic Agents on the DNA Sequence and Epigenetics in Plants

The mutagenesis and carcinogenesis affecting DNA sequence and chromatin structure through Ethyl methane sulfonate (EMS) mutagenesis and associations with epigenetic changes have been explained (Yan et al., 2021) in rice. Whole-genome and re-sequenced data congregated from 52 rice EMS mutants facilitated mutation for altering DNA sequences and the probable linkages along with chromatin composition. Single nucleotide polymorphic sites (SNPs) along with genomic facets related to EMS anchored mutagenesis prejudices were unraveled. EMS, equated with natural SNPs available in the Rice 3K project, displayed a liking to G/C sites with flanking motifs higher in GC amounts. Efficacies of EMS mutagenesis and constituents of local dinucleotides along with trinucleotides were having associations. The prejudiced allocation of EMS indicted SNPs were affiliated in a positive direction with transposable element quantities, CpG numbers, and suppressive epigenetic markers but linked in the negative direction with active epigenetic markers and gene (s) displaying the euchromatin marker DNase I hypersensitive sites. Another example through which mutations created epigenesis was presented with *Arabidopsis thaliana* mutants originated straightway by changes in DNA methylation affecting transcription of the gene. The late-flowering mutant flowering Wageningen (FWA) created by ectopic demonstration of the FWA gene enciphers a homeodomain-containing transcription facet. In wild type, the escalating region of FWA is methylated DNA and FWA is not produced in vegetative tissues. When this methylated DNA is ousted from the ddm1 mutant, the FWA is noticed in vegetative tissues and causes late flowering. This late-flowering phenotypic form is also noticed in the mutant suggesting that silencing of FWA mainly depends on CG methylation (Soppe et al., 2000). In another recent study done at Indian Agricultural Research Institute, New Delhi, we treated Pusa 372- a high-yielding, and widely grown chickpea cultivar having moderate resistance/tolerance to major diseases with 0.3% EMS for 6 h at room temperature and found mutants with phenotypic variations for the increased number of pods (unpublished).

### Role of Tissue Culture on the DNA Sequence and Epigenetics in Plants

Tissue culture techniques are soul for any alteration at the genome level in crops. These techniques are also influenced its epigenome. The high-resolution maps of DNA methylation made in rice lines have reported that the regenerated plants have less methylation than control plants. The alterations were relatively over-represented around the promoter sequences of genes and affect gene expression. Critically, the plants’ offshoots also inherit the changes in methylation level (Hume et al., 2013). These aftermaths partly narrate the processes of somaclonal diversities that push forward epigenetic changes in the plants.

### Role of RNAi Techniques in Genome Epigenesis in Plants

Gene silencing through RNA inference (RNAi) approach was widely used to better understand the gene function in plants. Repression of translation was achieved through post-transcriptional modifications using RNAi. Interestingly, many of the factors that mediate post-transcriptional silencing via RNAi also contribute to transcriptional gene suppression (Fire et al., 1998). In plants, RNA viruses were observed to guide DNA methylation of homologous genes along with introducing multiple transgene copies resulting in silencing (Napoli et al., 1990; Van der Krol et al., 1990).

### Role of CRISPR/Cas9 in Genome Editing in Plants

The ribonucleoproteins (RNPs), containing Cas9 enzyme along with single-guide RNA (sgRNA) are successfully delivered using transformation methods or nanoparticle-based delivery approaches. The prime enzyme 4-coumarate ligase (4CL) involved in phenylpropanoid metabolism and responsible for the lignin biosynthesis process governs the congregation of lignin in distress stages. The 4CL along with the gene Reveille 7 (RVE7) linked with drought tolerance has been used for protoplast targeted mutagenesis in chickpeas. For the first time, chickpea protoplast was used as a transfection platform for CRISPR/Cas9-based genome editing in chickpeas (Badhan et al., 2021). The outcomes showed efficient editing got for the RVE7 gene *in vivo* compared with the 4CL gene.

Understanding genomic activities need site-specific modification at the loci *via* targeting systems (Papikian et al., 2019). Limited approaches for the desired manipulation of the epigenome present in plants were observed and adopted by the Cas9-system to design desired gene activation and DNA methylation in Arabidopsis.

## Connotations of Epigenetics and Epigenomics in Chickpea Improvement

Epigenetics and epigenomics display certain connotations as useful immense potentials along with numerous threats and challenges as stated by Springer and Schmitz (2017). Some of the potential connotations for utilization are summarized as given below.

### Resistance to Biotic and Abiotic Stresses

As we mentioned above, the role of epigenetics in disease resistance (Sen et al., 2017), cold tolerance (Rakei et al., 2015), drought, salinity tolerance (Khandal et al., 2017), and manipulating the epigenome may provide a promising breeding strategy to enhance yield, disease resistance, or adaptation for changing environmental conditions in chickpea, as shown below.

i. DNA methylation patterns in cultivated chickpeas to understand the regulation of gene expression in different organs (Bhatia et al., 2018)
ii. Drought and salinity resistance by an epigenetic mechanism in chickpeas (Sen et al., 2017)
iv. DNA methylation and epigenetics mechanism on physio-biochemical analysis of chickpea in response to cold stress (Rakei et al., 2015)
v. The epigenetic mechanism to heat stress in chickpeas (Chidambaranathan et al., 2016)
vi. Role of epigenetics in drought yield index (Sharma et al., 2019)

### Avoiding the Transgene Silencing in GM Crops

Transgene methylation and transcriptional gene mutations are directly correlated to each other (Matzke et al., 1989; Park et al., 1996) mainly because of the association between methylation of the coding sequence and post-transcriptional gene oppression (Ingelbrecht et al., 1994). Although, the latest proof shows that a merging process gleaned from RNA interference is primary to both processes (Matzke and Matzke, 2004). The intricate and meticulous designing of the transgene constructs and intense dissection of transformants at the molecular level are the prerequisites of an efficient technique to avoid transgene silencing (Dewilde et al., 2000). Two dominant classes of transgene silencing, the first one results in position effects (Matzke et al., 2000) and the second one is silencing phenomena or homology-oriented gene silencing, HDGS (Meyer and Saedler, 1996). Some examples reported in plants are tobacco, transgenic tobacco (*N. tabacum*) that are constructed ectopically over-express *AtMYB90v* (*Arabidopsis thaliana MYB 90*) promoter gene in association with regulating anthocyanin production in *Arabidopsis thaliana*. Transgenic tobacco overexpressing *AtMYB90* involved in anthocyanin biosynthesis showed siRNA-mediated silencing because of systemic acquired silencing (Velten et al., 2012).

### Evolution

Although epigenetics in multicellular organisms is the major mechanism for diversifications, with epigenetic motifs “reset” when organisms procreate, there were certain reflections of trans-generational epigenetic transmission, e.g., the phenomenon of para-mutation in maize (Brink, 1956). Epigenetic characters are multigenerational and eventually diminished over many generations. Then, there is a clear-cut maximum probability for explaining another aspect of evolution and adaptation. There are some speculations that the differential mutation rates associated with epigenetic features were taken as an advantage by the organisms that control the mutation rates of particular genes. Epigenetic changes have also been reflected to originate in reaction to environmental exposure; for example, epigenetic alterations are prevalent in inter-specific hybrids and polyploids. DNA methylation patterns after hybridization and/or polyploidization can be primarily changed by these re-patterning processes, as exemplified by studies in *Brassica, Arabidopsis, Triticum*, and *Oryza*. In these species, methylation-influenced AFLP assays provided widespread alterations in genomic methylation, including modifications in genes (Liu and Wendel, 2003).

A study unraveled DNA methylation systems in cultivated chickpea to explain the control of gene expression in variegated organs primarily by the methylating systems in leaf tissue of wild and cultivated chickpea. The results show a positive association of promoter hyper-methylation with increased transcript paucity through recognition of DMR of the genes governing flower development meant in cultivated chickpeas (Bhatia et al., 2018).

### Genetic Variability

Epigenetic patterns in plants, once instituted, can be transmitted through the inheritance of epialleles across many generations (Kakutani, 2002). Such transmittable epigenetic alleles can be assumed as a novel source of polymorphism and may reproduce new phenotypes. Evaluating the significance of methylated epialleles in crop breeding requires the genetic variability in the selected population for the degrees that methylating modes influence superior phenotypes and the extent to which methylation is statically transmitted. DNA methylation was first reported in regeneration studies of crown gall tumor events in which phenotypic variability and methylation of T-DNA were linked (John and Amasino, 1989). The most interesting evidence suggests that a substantial proportion of somaclonal variation might be because of diverse, pre-existing epigenetic states’ result in the regeneration of individual somatic cells (Neuhuber et al., 2005)*.*

Epigenetic initiation of DNA elements through transposable elements with *Arabidopsis* suggests epigenetic modifications may also be intricated in cytogenetic instability through changes of heterochromatin, and as a basis of phenotypic diversity through the modulation of gene functionalities (Kaeppler et al., 2000).

Epigenetics plays an important role in somaclonal variation, and chromatin modulation plays an important role in gene expression regulation and genome activities (Azizi et al., 2020). Some epigenetic modifications that induced intergenerational distress memory resistance in crop plants in addition to as presented in Table 5 are as below.

a) Chickpea*-*drought water and osmotic stress (Elkoca et al., 2007; Kilian et al., 2007)
b) Canola–Salt/drought, seed priming with NaCl, increased energy efficient utilization, and PGPR for halo-tolerant plant (Farhoudi et al., 2007)
c) Sugarcane–Drought/salinity, NaCl, and PEG-primed seeds (Marcos et al., 2018a)

**TABLE 5:** Intergenerational stress memory resistance development in crop plants through epigenetic modifications.

Crop species	Stress resistance	Treatment/Pathway	References
Chickpea (*Cicer arietinum*)	Drought	Water and osmotic stress	Elkoca et al. (2007) and Kaur et al. (2008)
Mung bean (*Vigna radiate*)	Drought/salinity	Halopriming of seeds with NaCl and PEG	Jisha and Puthur (2014)
Alfalfa (*Medicago sativa*)	Drought	Seed osmotic treatment with PEG	Mouradi et al. (2016)
Cowpea (*Vigna unguiculata*)	Drought	Water, osmotic, and hormonal seed stress	Eskandari and Kazemi (2011) and Boucelha and Djebbar (2015)
Arabidopsis (*Arabidopsis thaliana*)	Drought/salinity/biotic stress	β-amino-butyric acid, hyperosmotic priming of seedlings	Slaughter et al. (2012) and Sani et al. (2013)
Soybean (*Glycine max*)	Drought/salt	Indole acetic acid and NaCl stress on seedlings induced long non-coding RNAs and DNA methylation	Umezawa et al. (2000) and Chen et al. (2019)
Mung bean (*Vigna radiate*)	Drought/heavy metals	Indole-3-butyric acid	Li et al. (2018b)

### Heterosis

Heterosis is the superiority of the F_1_ hybrid phenotype over its parents. The phenomenon has been exploited extensively in agricultural breeding for decades and, despite its commercial impact; it has also improved crop performance tremendously. However, knowledge of the molecular basis underlying heterosis remains incomplete. Most studies have focused on finding genetic explanations, resulting in the classical dominance and overdominance models of heterosis (East, 1908; Shull, 1908; Bruce, 1910; Jones, 1917). Identification of better hybrids through the utilization of hybrid vigor in chickpea by assessing seven F1 hybrids inclusive of nine cultivars was executed (Ghfaffar et al., 2015). Paramount heterosis along with heterobeltiosis for plant height and subsidiary branches were observed in the cross K0014–10 × K0066-10, however, cross K0019–10 × K0031-10 performed the highest heterosis along with heterobeltiosis for principal branches and seed number plant^−1^, the cross K0014–10 × K0052-10 reflected the highest heterosis and heterobeltiosis with 33.18 and 30.84% for 100 seed weight and 97.37 and 76.47% for seed yield plant^−1^, discretely. Broad sense heritability for various characters observed varied from 63.14 to 77.18%. Remarkable heterosis, heritability, and genetic advance were recorded for pod number plant^−1^ that could be employed for identifying best segregate from crosses K0031–10 × K0052–10, K0019-10 × K0026-10, and K0019–10 × K0031-10. Best hybrids from the observation could be employed for the betterment of multiple traits by identifying single plants for varied characteristics.

### Hybridization and Epigenetic/Epigenomic as Predictive Markers for Hybrid Performance

Molecular profiling of superior hybrids reflected that their epigenomes are substantially remodeled to their parental lines, leading to epigenetic states that deviate from the expected mid-parent values. Extensive remodeling has been observed at the level of DNA methylation in *Arabidopsis* (Zhang et al., 2016), rice (He et al., 2010), pigeon pea (Zhang et al., 2006), broccoli (Li et al., 2018a), and rapeseed (Shen et al., 2017). It occurs either at regions where parents are differentially methylated (DMRs).

## Potential Challenges to Epigenetics and Epigenomics

Irrespective of the immense potential of epigenetics for opening new avenues for utilization in crop improvement programs, there are certain challenges and threats as stated below.

A. Determination of Epigenetic State: The first challenge is to clearly define the basis of an epigenetic state. Whereas a DNA sequence is simply defined by the order of the four bases (A, C, G, and T), the exhaustive list of components that define given chromatin or epigenetic state is yet to be established. These components include methylation of cytosines and adenines, mono-, di- or tri-methylation, acetylation, phosphorylation, ubiquitination, etc of histones at various positions (e.g., H2AK119, H3K4, H3K9, H3K27, H3K36, etc) and long or short ncRNAs produced in cis or trans.
B. Determination of Chromatin Stability: The second important challenge is to define the stability of given chromatin. Three major levels of stability can be distinguished, as given below:
Transient Chromatin States: These chromatin states are specific to a different cell or established in immediate response to biotic or abiotic stress, and do not persist after the stimulus is removed.
Metastable Epigenetic States: These epigenetic states are started by specific stress or environmental inductions and can persist across multiple cell divisions after induces.
Inherited Epigenetic States: These epigenetic states are transmitted across multiple generations and are typically correlated with TEs or other repeat sequences. It is still unclear what role the environment plays in initiating or erasing these states.
Gene and environmental interactions as epigenetics are influenced by the environment and sometimes it leads to non-stable variations.
Epigenome sequencing methods are not well established.

## Future Perspective: Exploring Epigenomics as a New Kind of Plant Breeding

Epigenetics has immense potential for opening new avenues for crop improvement programs stated as followings:

a) Variation: Considerable natural variability in the DNA methylation process exists within many plant species.
b) Stable Inheritance: Variability in DNA methylation can originate through processes and clonal propagation can propel epigenetic alleles.
c) Epigenome Engineering: New epigenome editing tools provide broader opportunities to create new epiallelic variants by altering the methylation of DNA or other modifications at the chromosome level. These tools can be used for crop improvement through epigenome engineering.
d) Emerging New Technologies: The development and application of methods for widespread epigenome profiling and engineering may generate new avenues for using the full potential of epigenetics in crop improvement.
e) Sources for Biotic and Abiotic Resistance: Epigenetics has become an important research focus at a time when rapid environmental changes are occurring. They enhance fitness extremely rapidly without depending on the slower process of natural selection through changing DNA-encoded genetic variants in plant populations.
f) Time and Cost-Effective: Epigenetics introduces as a time- and cost-effective tool in plants as a source of resistance against new future abiotic and biotic stresses.
g) Public and Producer’s Acceptance: Successful implementation of all crop enhancement approaches at the DNA level requires support from the public and government and epigenome editing does not change the genome sequence might ease the challenges of public acceptance for epigenetically modified products.
h) Equilibrium among important agronomic traits: Plants use epigenetic variation to reprogram their transcriptome in a precise and timely manner to maintain equilibrium amongst important agronomic traits.

## Conclusion

Climate change is altering the predominance of varied environmental situations, and improved distress tolerance has become a primary breeding goal in chickpeas. *In vivo* situations, crops are usually concomitantly opposed by diverse biotic and abiotic distresses. Hence, grasping possible processes responsible for the occurrence of stresses has become a necessity for stable crop productivity. The epigenetic mechanisms play an important role in a classical plant breeding program, mainly by genetic heritability, hybrid vigor, plant-environment interactions, abiotic and abiotic stress tolerance, and yield stability performance of crop plants. A better and deep insight into epigenetic mechanisms might facilitate plant breeders in creating novel and more super crop varieties that can include natural phenotypic diversity. It is a very interesting fact that the environmental shielding effects of epigenetics are directly associated with those genes that play a very important role in the regulation of plant growth and yield in chickpeas and other crops. Furthermore, understanding the molecular bottom of trans-generational epigenetic transmission puts forward the development of epialleles identified for specific environmental status through combined and multidisciplinary efforts of researchers and targeted epigenetic modifications in genes of interest. Thus, epigenomics, either as exploitation of existing epigenomic variability or alteration of the epigenome, can complement conventional plant breeding to ensure global food security and sustainable agriculture.

## Data Availability

The original contributions presented in the study are included in the article/Supplementary Material, further inquiries can be directed to the corresponding author.
